# FDA Approvals of Biologics in 2022

**DOI:** 10.3390/biomedicines11051434

**Published:** 2023-05-12

**Authors:** Alexander C. Martins, Fernando Albericio, Beatriz G. de la Torre

**Affiliations:** 1 School of Health Sciences, UAM, Universidade Anhembi-Morumbi, São Paulo 03101-001, Brazil; 2 School of Chemistry and Physics, University of KwaZulu-Natal, Durban 4001, South Africa; 3 CIBER-BBN, Networking Centre on Bioengineering, Biomaterials and Nanomedicine, Department of Organic Chemistry, University of Barcelona, 08028 Barcelona, Spain; 4 KRISP, College of Health Sciences, University of KwaZulu-Natal, Durban 4001, South Africa

**Keywords:** monoclonal antibodies, antibody-drug conjugate, first approval, FDA, Biologics, anacaulase, eflapegrastim, enzymes, orphan drug

## Abstract

The year 2022 witnessed the control of the COVID-19 pandemic in most countries through social and hygiene measures and also vaccination campaigns. It also saw a decrease in total approvals by the U.S. Food and Drug Administration (FDA). Nevertheless, there was no fall in the Biologics class, which was boosted through the authorization of 15 novel molecules, thus maintaining the figures achieved in previous years. Indeed, the decrease in approvals was only for the category of small molecules. Monoclonal antibodies (mAbs) continued to be the drug class with the most approvals, and cancer remained the most targeted disease, followed by autoimmune conditions, as in previous years. Interestingly, the FDA gave the green light to a remarkable number of bispecific Biologics (four), the highest number in recent years. Indeed, 2022 was another year without the approval of an antimicrobial Biologic, although important advancements were made in targeting new diseases, which are discussed herein. In this work, we only analyze the Biologics authorized in 2022. Furthermore, we also consider the orphan drugs authorized. We not only apply a quantitative analysis to this year’s harvest, but also compare the efficacy of the Biologics with those authorized in previous years. On the basis of their chemical structure, the Biologics addressed fall into the following classes: monoclonal antibodies; antibody-drug conjugates; and proteins/enzymes.

## 1. Introduction

In 2022 (also referred to as “this year” herein), after two years fighting the COVID-19 pandemic, the world finally saw a decrease in infections, hospitalizations, and deaths by SARS-CoV-2. This turnaround was attributed to a variety of safety measures adopted by governments and to vaccination campaigns. However, there was also a decrease in the total number of drugs approved by the U.S. Food and Drug Administration (FDA) in 2022 (37 vs. an average of approximately 50 in recent years). This fall could be linked to the fact that most pharmaceutical companies focused their resources on tackling the COVID-19 pandemic to the detriment of their normal activities.

In terms of total drug approvals (New Chemical Entities (NCEs) and Biologics) between 2015 and 2022, the lowest numbers were registered in 2016 (22) and 2022 (37) [1,2]. On the other hand, regarding Biologics, 2022 had the highest percentage of approvals (40%), thereby indicating that the presence of this class is fully stabilized in the pharmaceutical industry. In recent years, Biologics have accounted for less than 30% of the total drugs approved (Table 1).

We highlight new drugs and their efficacy, mechanisms, and targets, and compare them to the Biologics already on the market. We also undertake a quantitative and qualitative analysis of the approvals and discuss the Biologics market. Of note, we did not include biosimilars in the analysis. We also reviewed clinical trials ongoing for all the Biologics mentioned herein, testing them for diseases other than their primary targets approved, exposing the potential of these drugs to treat other diseases in the long term.

## 2. Analysis

Several factors, among them industry failures and/or political issues, can explain a drop in the number of authorizations. For example, 2016 registered a decrease in submissions and also an increase in the rejection rate of drugs by the FDA. Of note, 2016 was also an election year in the United States, with Donald Trump taking office in January 2017 [11,12]. The following election year in that country was 2020, with Joe Biden taking over in January 2021, and there was a completely different scenario in terms of the total number of drug approvals, as that year registered the second highest figure (53) in the period 2015–2022. Importantly, the World Health Organization (WHO) declared the COVID-19 pandemic in 2020. In response, the FDA, along with other organizations, devoted efforts to research, and accelerated approval and review processes, resulting in many Emergency Use Authorizations issued for vaccines and other drugs. This scenario also affected the authorization process of specific drugs such as Veklury^TM^ (Remdesivir-2020), for example, indicated to treat COVID-19 [5,13]. 

Although 2016 registered the lowest number of drug authorizations in that period, it witnessed the approval of two key antibacterial mAbs, namely Anthim^TM^ (oblitoxaximab), indicated to treat inhalation of *Bacillus anthracis* (anthrax), and Zinplava^TM^ (bezotoxumab), indicated to reduce recurrent infections of *Clostridum difficile* [9]. Oblitoxaximab and bezotoxumab were the only antibacterial mAbs approved in the period 2015–2022, and this fact gains relevance in the context of increasing antibacterial/antimicrobial resistance [12,14,15].

**Figure 1:**
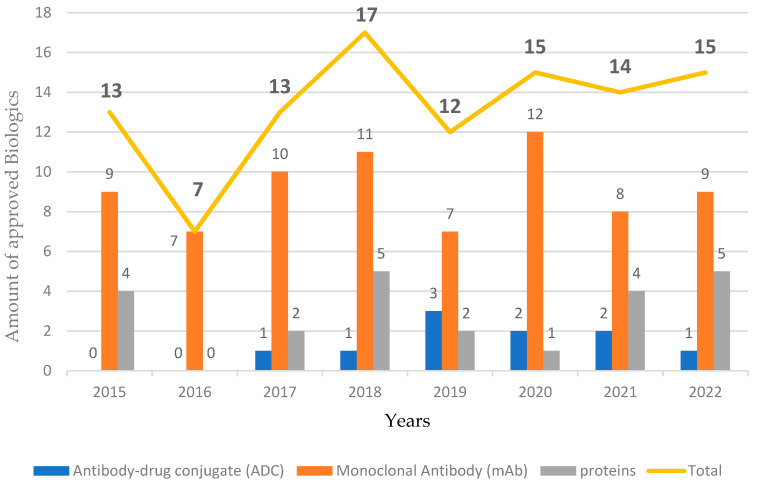
Biologics approved by the Food and Drug Administration (FDA) from 2015 to 2022 [1,3].

As seen in Figure 1, in 2022, the number of approvals of the three classes of Biologics hardly varied from the figures of previous years. Similarly, mAbs for cancer continued to be the therapeutic indication receiving the most approvals, with six Biologics, followed by autoimmune conditions, with four. The other conditions and diseases targeted by the Biologics authorized this year included eschar removal from thermal burns, aesthetic purposes, chemotherapy-induced neutropenia (CIN), eye disorders, and acid sphingomyelinase deficiency (ASMD).

Fifteen Biologics were approved in 2022 and mAbs continued to account for the majority of FDA approvals among them. The authorization of mAbs in 2022 was slightly higher than in 2021 (nine vs. eight, respectively), the same applied for proteins and enzymes, while there was one less ADC approval than in 2021. The authorization of a new mAb for Alzheimer’s Disease (AD) was expected in 2022, but it was finally approved in January 2023.

## 3. Orphan Drugs

All drugs must go through the pertinent development processes and subsequent approval and licensing process. However, the submission of a request seeking Orphan Drug Status is a completely different process and can be started by sending the required information by regular mail to the Office of Orphan Products Development at the FDA, emailing this information to the correct FDA address, or submitting it through the CDER NextGen portal [16]. As their name indicates, Orphan Drugs are intended to treat orphan diseases (rare conditions and diseases). These pose important and specific challenges in the development process, such as difficulties in clinical trials as a result of small patient populations, problems in the recruitment process, and a lack of knowledge of the disease. Furthermore, the concept of rare disease may vary from country to country. Despite these challenges, the pharmaceutical industry as a whole increasingly addresses the urgency of developing more treatment options for these kinds of diseases. In this context, the incentives provided by the FDA also drive greater resource allocation to these diseases. Annual growth in the development of Orphan Drugs is now expected [17,18].

Interestingly, as seen in Table 2 46% (seven) of all approvals in 2022 received Orphan Drug Status from the FDA. This is a considerable number given the difficulty faced by the pharmaceutical sector. The number of drugs to be awarded this status is almost half that of the new Biologics approved each year. Of note, enzymes are emerging as key approaches to tackle rare diseases and conditions. In this context, all the enzymes approved from 2015 to 2022 received Orphan Drug Status.

## 4. Therapeutic Indications and Mechanisms of Action

### 4.1. Cancer

Of the 15 Biologics approved in 2022, six were indicated for the treatment of a diversity of cancers (Table 3). Comparatively, we had four Biologics approved indicated for cancer approved in 2019, eight in 2020, and six in 2021 [1]. From 2019, there has clearly been a growth aimed at cancer in the Biologics market.

The first-in-class Biologic, namely the bispecific fusion protein Kimmtrak^TM^ (tebentafusp), which is intravenously administered, is the first drug to date specifically for the treatment of metastatic uveal melanoma in HLA-A-positive patients, leading the immune system directly to the cancer cell [19]. One arm of tebentafusp (anti-CD3 effector) binds to T lymphocytes, later dragging the T cell to the cancer cell. This immune cell must bind to glycoprotein 100 (gp100), which may be inside the tumor cell, and therefore needs to be presented to the tumor cell surface through the human leukocyte antigen (HLA). The other arm of tebentafusp (T-cell receptor arm) then targets gp100, binding to the melanoma cell and activating the T cell, which then kills the melanoma cell. Other Biologics have previously been approved for the treatment unresectable or metastatic melanomas, namely Yervoy^TM^ (ipilimumab) in 2011, Keytruda^TM^ (pembrolizumab) and Opdivo^TM^ (nivolumab) in 2014 [28], Tecentriq^TM^ (atezolizumab) in 2016 [1], and Opdualag^TM^ (relatlimab and nivolumab) in 2022. However, tebentafusp is the first Biologic indicated for metastatic uveal melanoma. This type of cancer is very different from other melanomas as it shows distinct patterns, pessimistic prognosis, and a high likelihood of metastasis [29]. These characteristics thus make Kimmtrak^TM^ an important breakthrough. Overall Survival (OS) was the main measure found in the literature for Kimmtrak^TM^, with a median OS of 21.7 months vs. 16 months for the control group [19,29]. For Opdualag^TM^, which is intravenously administered, the main measure found was Progression-Free Survival (PFS) vs. nivolumab alone, with a PFS of 10.1 months for Opdualag^TM^ vs. 4.6 months for nivolumab [21].

As shown in Ref. [30], the combination of mAbs such as Opdualag^TM^ (nivolumab and relatlimab), which was approved this year, offers the interesting advantage of simultaneously targeting multiple pathways. Opdualag^TM^ provides a first-in-class mechanism of action by carrying two fully human mAbs, the first one targeting LAG-3 receptors and the second one PD-1 receptors, thereby increasing T-cell activation [21]. Bispecific mAbs can also target more than one pathway. However, three distinct mAbs can be combined, as is the case of Phesgo^TM^, in which all the mAbs target the glycoprotein (GP) of *Zaire ebolavirus* but in distinct ways. In this regard, between 2015 and 2022, only three combinations of mAbs have received approval, namely the aforementioned Phesgo^TM^ (pertuzumab, trastuzumab, and hyaluronidase) for Ebola vírus, Inmazeb^TM^ (atoltivimab, maftivimab, odesivimab) to treat early or metastatic breast cancer, both approved in 2020 [1], and Opdualag^TM^, which received authorization this year [21].

Of note, the last fusion protein approved by the FDA was in 2018, with tagraxofusp, indicated for the treatment of blastic plasmocytoid dendritic cell neoplasm [1]. While the two cancer drugs tagraxofusp and tebentafusp received Orphan Drug Status, tebentafusp is the first bispecific fusion protein to get the green light to date.

Lunsumio^TM^ (mosunetuzumab), a humanized bispecific mAb, has received accelerated approval from the FDA this year. Indicated to treat a type of non-Hodgkin’s lymphoma (relapsed or refractory follicular lymphoma (FL), it presented an Objective Response Rate (ORR) of 80% in clinical trials, with 60% of patients presenting a Complete Response (CR) [27]. Patients affected by follicular lymphoma (FL) have very few treatment options when it comes to Biologics. The other treatment option for this condition is rituximab, which was approved in 1997 and was the first mAb for cancer patients. Its therapeutic indications include FL. In comparison with the new bispecific mosunetuzumab, rituximab has an ORR of around 50% and a CR of 6% [31]. In 2017, we saw the approval of a reformulated Rituxan Hycela^TM^ (rituximab) with the addition of the human enzyme hyaluronidase, which is subcutaneously administered [32]. In this regard, no other mAb has been authorized since Rituxan Hycela^TM^, which is indicated to FL; it has taken five years for a new mAb for this disease to come onto the market.

Importantly, from 2015 to 2022, the FDA authorized only four bispecific antibodies, namely emicizumab (2017), amivantamab (2021), faricimab (2022), and mosunetuzumab (2022) [27,33,34,35].

Imjudo^TM^ (tremelimumab) was approved for cancer this year, intravenously administered, indicated for unresectable hepatocellular carcinoma (uHCC) [23]. It is a mAb whose mechanism works by blocking CTLA4, thus stopping the interaction of ligands with the cytotoxic T-lymphocyte-associated antigen 4. The previous cancer Biologic indicated for uHCC to get the green light was Tecentriq^TM^ (atezolizumab)(2016). This Biologic is also a mAb but, in contrast to tremelimumab, it acts by blocking PD-L1 [1]. For uHCC, both drugs are indicated to be used in combination with other mAbs, namely atezolizumab + bevacizumab, and tremelimumab + durvalumab.

In clinical trials, tremelimumab combined with durvalumab, a PD-L1 blocker, demonstrated higher OS (16.43 months vs. control group 13.72 months) and also a better ORR (20.1 vs. 5.1, respectively) [36].

In another advancement by Janssen Biotech Inc., Tecvayli^TM^ (teclistamab), indicated for relapsed or refractory multiple myeloma (MM), was approved in 2022. Importantly, from 2015 to 2021, the FDA authorized five other Biologics for this disease, namely: Darzalex^TM^ (daratumumab) and Empliciti^TM^ (elotuzumab), both in 2015, and Darzalex Faspro^TM^ (daratumumab and hyaluronidase), Sarclisa^TM^ (isatuximab), and the ADC Blenrep^TM^ (belantamab mafodotin), all three in 2020 [1]. Of note, all the Biologics for MM hold Orphan Drug Status.

Belantamab mafodotin (approved in 2020) binds to the B-cell maturation antigen (BCMA), and therefore, has a similar mechanism of action to that of the novel teclistamab. However, the latter is the first bispecific mAb to treat MM. It binds to BCMA and also to CD3 receptors [24]. In clinical trials, teclistamab showed a good ORR, with 40% of the patients presenting a CR [37,38,39].

Intravenously administered, Elahere^TM^ (mirvetuximab soravtansine) was the antibody-drug conjugate (ADC) of 2022 to be approved (fast-track process) by the FDA [40]. This ADC is a FRα-directed (folate receptor alfa) chimeric mAb that targets epithelial ovarian cancer, which has high expression of FRα. When internalized, Elahere^TM^ releases its small molecule (DM4), a microtubule inhibitor, after cleavage of its disulfide linker, unleashing apoptotic cell death. The anti-tubulin agent DM4 is an analog of maytansine, which was last found, before 2022, almost one decade ago in another ADC Kadcyla^TM^ (rastuzumab emtansine) [30]. DM4 is genotoxic, it confers risk to pregnant women, and it is a potent CYP3A4 substrate. Patients treated with DM4 must be closely monitored [25]. From 2015 to 2021, nine ADCs were approved by the FDA [1] and Elahere^TM^ is the tenth of this class.

Regarding efficacy, in a single-arm trial, Elahere^TM^ demonstrated an ORR of 31.7% and a DOR of 6.9 months, but further research is still ongoing [40,41].

#### Ongoing Clinical Trials for the New Biologics for Cancer

There are trials ongoing for tebentafusp (phase 1b/2) to test this Biologic in metastatic cutaneous melanoma, but in combination with other Biologics (durvalumab and/or tremelimumab), and also tebentafusp alone in advanced non-uveal melanoma, with no results posted yet [42]. Regarding trials for nivolumab and relatlimab to potentially treat diseases other than its primary target, there are trials ongoing to test it in metastatic or unresectable chordoma and [43], a phase 2 trial to test it in advanced microsatellite stable (MSS) colorectal cancer [44], a phase ½ trial to test its effectiveness in liver cancer [45], and interestingly, just like Kimmtrak^TM^ (tebentafusp) mentioned earlier in this paper, whose therapeutic indication is metastatic uveal melanoma (MUM), the first treatment to date specifically for MUM, there is a phase 2 trial ongoing to test nivolumab and relatlimab for MUM [46]. A combination of mAbs such as Opdualag^TM^ carries great potential for repurposing and exploiting new targets/diseases; unfortunately, there are no results posted yet regarding the ongoing trials mentioned.

Tremelimumab is being tested for bladder cancer, with a completion study date in 2026 [47]. It is currently only approved for adult patients, but ongoing studies were found to test tremelimumab in combination with durvalumab in pediatric patients with solid tumors and hematological malignancies [48], and a phase 1 study with a completion date in 2024 for metastatic melanoma [49].

Mirvetuximab soravtansine is being tested as a first-line treatment for triple-negative breast cancer [50], and in combination with pembrolizumab as a new option for endometrial cancer in a phase 2 study, expected to be completed in 2025 [51].

There are trials with mosunetuzumab for four other conditions: reduction of the tumor with mosunetuzumab in combination with polatuzumab vedotin for refractory, relapsed, or aggressive non-Hodgkin lymphoma in a phase 2 study [52]; a phase 1 study to test mosunetuzumab to treat B-cell lymphoma after replacement of patient’s stem cells by autologous stem cell transplant [53]; and a phase 1 study assessing the efficacy of mosunetuzumab in relapsed or refractory chronic lymphocytic leukemia (CLL) [54]. All of these three studies have a completion date expected in 2027. There is still a phase 1 study testing mosunetuzumab for systemic lupus erythematosus, with a completion date expected in 2024 [55]. None of these studies have posted results yet. Regarding teclistamab, ongoing trials were not found for a disease other than multiple myeloma.

### 4.2. Autoimmune Conditions

The second type of disease most targeted by the approved Biologics in 2022 is autoimmune conditions (Table 4).

Spevigo^TM^ (spesolimab) has been approved this year to treat generalized pustular psoriasis (GPP), a rare and autoinflammatory condition that can strike both children and adults, affecting more Asians than other population groups [60]. To date, there is no standard treatment specifically for GPP, therapeutic strategies being limited to the use of synthetic drugs and Biologics previously authorized for moderate and severe plaque psoriasis, which have a poor outcome in GPP. As other Biologics indicated to treat plaque psoriasis or psoriatic arthritis have distinct interleukin receptors as targets (i.e., IL-17R), spesolimab brings a new mechanism of action by binding to IL-36R (interleukin-36 receptor), thereby inhibiting IL-36 from binding to IL-36R [20], since GPP seems to have a singular mechanism in its pathogenesis involving the IL-36R [60]. Although further research is needed on this subject, current studies support the efficacy of anti-IL-36R therapy in GPP [60,61,62].

Briumvi^TM^ (ublituximab) (2022) is intravenously administered and it is indicated to treat relapsing forms of multiple sclerosis. Its mechanism of action is like that of Ocrevus^TM^ (ocrelizumab), the previous mAb for multiple sclerosis approved by the FDA, in 2017. These two drugs bind to CD-20 on B-cells, both pre-B cells and mature B cells, thereby unleashing cell lysis [59,63]. Prior to ocrelizumab, the FDA had only approved Zinbryta™ (daclizumab) (2016) [1], which acts by binding to a subunit of IL-2 receptors, namely CD-25. Between 2015 and 2022, only these three mAbs received the green light for this condition. 

Ublituximab is a chimeric mAb, and from 2015 onwards, we have seen a really low number of chimeric mAb approvals by the FDA, namely: Unituxin^TM^ (dinutuximab) in 2015; Anthim^TM^ (obiltoxaximab) in 2016; Rituxan Hycela^TM^ (rituximab and hyaluronidase) in 2017; Sarclisa^TM^ (isatuximab) and Margenza^TM^ (margetuximab), both in 2020; and none of them are for multiple sclerosis [1], making ublituximab the first chimeric mAb for MM.

In trials, ublituximab has been demonstrated to be superior to an orally administered medication (teriflunomide) in the two endpoints evaluated. In the primary endpoint in trial I, the Annualized Relapse Rate (ARR) reported was 0.08 for ublituximab vs. 0.19 for teriflunomide, and in the same endpoint in trial II, it was 0.09 for ublituximab vs. 0.18 for teriflunomide. In the secondary endpoint in trial I, the average number of gadolinium-enhancing lesions was measured at 0.02 for ublituximab vs. 0.49 for teriflunomide 0.49, and in trial II it was 0.01 for ublituximab vs. 0.25 for teriflunomide, demonstrating lower rates and fewer lesions in the magnetic resonance imaging [64].

Tzield^TM^ (teplizumab) binds to its target CD3 and patients with Stage 2 type 1 diabetes (T1D) can benefit from a delay in the onset of Stage 3. This is a first-in-class and unique treatment that can deactivate certain immune cells involved in T1D. The efficacy of teplizumab in delaying the onset of Stage 3 T1D has been demonstrated in trials. Indeed, the primary measure was time from randomization to the diagnosis of Stage 3 T1D. It was observed that 19.8 (45%) of the patients (of a total of 44) receiving teplizumab had a later diagnosis of Stage 3 TID than the placebo group [57,58,65]. Teplizumab is intravenously administered and it is one of the few Biologics authorized in 2022 for both adult and pediatric patients.

Another important advancement in autoimmune diseases this year is the first-in-class Enjaymo^TM^ (sutimlimab), which is intravenously administered. It is indicated to decrease the need for red blood cells (RBCs) transfusion in cold agglutinin disease (CAD); CAD is a rare condition characterized by the destruction of RBCs in cold temperatures. Sutimlimab also brings a new mechanism of action by binding to the complement protein component 1, inhibiting the complement pathway [56,66]. In a clinical trial, more than half the patients positively responded to sutimlimab by increasing hemoglobin and no RBC transfusion was required after five weeks of treatment, and they reported decreased fatigue [67,68].

From 2015 to 2021, the FDA approved 13 Biologics for autoimmune conditions; as such, this is the second disease category to receive the most authorizations after cancer [1]. This year, we have seen four new Biologics added to this category.

#### Ongoing Clinical Trials for the New Biologics for Autoimmune Conditions

Boehringer Ingelheim is conducting studies to test spesolimab in other conditions. There are trials ongoing to test the efficacy of spesolimab for palmoplantar pustulosis (PPP) in a phase IIa study, with results supporting its efficacy vs. the placebo, but there are still trials ongoing to keep testing for PPP [69,70]. In 2024, a phase 2 study is expected to be completed to test the efficacy of Spesolimab in hidradenitis suppurativa (HS) [71], ulcerative colitis (UC) [72], an improvement of the narrowing of the small bowel in Crohn’s disease patients [73], and there are also studies ongoing to test it in atopic dermatitis (AD) and other conditions whose mechanism is similar to those that can cause HC, UC, or AD [74,75].

Ublituximab is being tested in combination with umbralisib for proggressive CLL in a phase 2 trial, and in a phase 1 and 2 study testing tazemetostat in combination with umbrasilib and ublituximab to treat relapsed or refractory follicular lymphoma [76,77]; no results have been posted yet for either study. Regarding teplizumab and sutimlimab, ongoing trials were not found for diseases other than the primary authorized ones described in the Prescribing Information.

### 4.3. Aesthetic

Daxxify^TM^ (daxibotulinumtoxin A) was the Biologic for aesthetic purposes approved by the FDA in 2022 (Table 5) and it is administered by intramuscular injection. It is found in the literature as an advancement, considering past decades of the hegemony of Botox^TM^ (onabotulinumtoxin A) to treat glabellar lines. Daxibotulinumtoxin A shows promising results and greater internalization of the neurotoxin, and clinical trials have demonstrated significant differences in response rate and also a longer period of effect for this Biologic. Patients in this trial also showed a better response to this Biologic than to the placebo [78,79,80]. The mean duration of the effects of daxibotulinumtoxin A in clinical trials is around 24 weeks, while for onabotulinumtoxin A it is around 19 weeks [79].

The IGA-FWS (Investigator Global Assessment-Facial Wrinkle Severity) and Global Aesthetic Improvement Scale (GAIS) were used to assess the results. Participants in the trial using 40 U of daxibotulinumtoxin A obtained between a 1 and 2 point improvement in glabellar lines, on both scales, over those using 20 U of onabotulinumtoxin A [79,82]. 

Before 2022, Jeuveau^TM^ (prabotulinumtoxin A) was the last Biologic authorized for aesthetic purposes (2019) [1]. Prabotulinumtoxin A and onabotulinumtoxin A presented similar outcomes in a 3-month study evaluating their effect on crow’s feet. The main measure of efficacy for prabotulinumtoxin A vs. onabotulinumtoxin A was mean onset of action (3.81 days for prabotulinumtoxin A vs. 3.47 days for onabotulinumtoxin A) and time to peak effect (9.58 days for prabotulinumtoxin A vs. 11.11 days for onabotulinumtoxin A). The secondary measure was the duration of action (11.11 weeks for prabotulinumtoxin A vs. 11.22 weeks onabotulinumtoxin A) [83]. The literature is still lacking data comparing the novel daxibotulinumtoxin A with prabotulinumtoxin A for the treatment of glabellar lines.

### 4.4. Eye Disorders

There have been two important drug advancements for eye disorders in less than three years. In this regard, back in 2019, the single-chain fragment variable (scFv) Beovu^TM^ (brolucizumab), which inhibits three isoforms of VEGF-A, received the green light from the FDA to treat neovascular (Wet) age-related macular degeneration (nAMD) [84]. Two years later, in January 2022, Vabysmo^TM^ (faricimab) (Table 6) was also approved for eye disorders such as nAMD and diabetic macular edema (DME). In the context of eye disorders, there is also ranibizumab, which was first approved in 2006 for nAMD, DME, and macular edema following retinal vein occlusion (RVO), and Eylea^TM^ (aflibercept), authorized in 2011 for nMAD. In clinical trials, the main measure of which was a change in Best-Corrected Visual Acuity (BCVA), brolucizumab outperformed aflibercept in minor endpoints and was non-inferior in primary endpoints, and it showed a higher remission of retinal thickness when compared to ranibizumab [85].

In contrast to brolucizumab, faricimab is a bispecifc humanized antibody [3,35]. The small size of the immunoglobulin fragments mechanism found in brolucizumab and its drug delivery features are important characteristics for Biologics, and these are also seen as important characteristics in bispecific mAbs, such as faricimab, whose mechanism is to inhibit two pathways, enhancing the fight against many diseases. Faricimab exerts anti-vascular endothelial growth factor-A (VEGF-A) and anti- angiopoietin-2 (Ang-2) activity [86]. In clinical trials, faricimab demonstrated similar outcomes in the same measure (BCVA) and anatomic improvement when compared to brolucizumab. However, further research is required [87].

#### Ongoing Clinical Trials for Faricimab

Regarding the potential of faricimab to treat other conditions, in this year (2023), a phase 2 trial has begun for faricimab to test non-proliferative diabetic retinopathy, but no results have been posted yet [88]. This year, two phase 3 trials are expected to be completed to test faricimab in macular edema due to hemiretinal vein occlusion, retinal vein occlusion, and central retinal vein occlusion [89,90]; no results have been posted yet for those studies as well.

### 4.5. Enzymes and Proteins

Three out of the fifteen Biologics to get the green light in 2022 fall into the class of proteins and enzymes, as found in Table 7. We discuss the results of the efficacy of these new Biologics compared the ones approved in previous years.

Xenpozyme^TM^ (olipudase alfa) was the first enzyme to be approved by the FDA in 2022. It is a replacement therapy indicated to treat a rare disease named acid sphingomyelinase deficiency (ASMD) (also known as Nieamann-Pick Disease) [91,92]. The deficiency of acid sphingomyelinase (ASM) leads to the accumulation of sphingomyelin and other lipids, which can cause involvement of the central nervous system (CNS), hepatosplenomegaly, and/or lung impairment. There are two types of ASMD, type A and type B. The former causes hepatosplenomegaly and CNS impairment, while type B leads to hepatosplenomegaly, and liver and lung impairment, and it may not present CNS disruption [95,96]. In clinical trials, olipudase alfa demonstrated improved clinical symptoms, including enhanced platelet counts, a reduction in liver and spleen volume, and a greater lung diffusing capacity, and it also cleared sphingomyelin from tissues [97,98].

Given the difficulty in managing neutropenia in some cancer treatments, Rolvedon^TM^ (eflapegrastim), which has been approved this year, is an important innovation. In this regard, Biologics for chemotherapy-induced neutropenia (CIN) started in 1991 with filgrastim, followed by pegfilgrastim in 2002. However, since then, the industry has struggled to develop a new Biologic other than biosimilars for CIN. Eflapegrastim has the addition of an Fc Fragment of a human IgG4 [93], which extends its half-life and increases its absorption by the bone marrow. In clinical trials, eflapegrastim demonstrated non-inferior efficacy in reducing neutropenia compared to pegfilgrastim at a reduced dose of G-CSF (Granulocyte-Colony Stimulating Factor); 3.6 mg and 6.0 mg, respectively, administered in all four cycles. Furthermore, the safety profiles of these two drugs are similar [99,100,101].

In 2022, nexoBrid^TM^ (anacaulase) was the only Biologic of topical administration approved with a distinct therapeutic indication: eschar removal in adults with full- or partial-thickness thermal burns. However, it still has significant limitations for the treatment of electrical and chemical burns, or burns to the face and genitalia [94]. Schar removal is a procedure that helps to better manage the wound and wound closure, and when eschar removal occurs in the first hours it can reduce bacterial growth and days of hospitalization [102]. Of note, no Biologic for this indication has been approved by the FDA in recent years.

#### Ongoing Clinical Trials for Eflapegrastim

Spectrum Pharmaceuticals Inc. is testing eflapegrastim for other conditions: pediatric participants with solid tumors or lymphoma and treated with myelosuppressive chemotherapy [103], to compare the effect of eflapegrastim on the duration of neutropenia in patients with early-stage breast cancer [104], but there are still trials to be carried out. No studies were found for anacaulase and olipudase alfa for diseases other than the primary ones described in the Prescribing Information.

## 5. Discussion

The quantitative aspect of total drug approvals by the FDA in 2022 could lead to some concern as it ranks as the second year with the lowest number [30]. However, as seen in 2016, factors such as fewer submissions and/or an increase in rejections by the FDA [11,12] may have occurred. Possible political influences should also be considered, including the end of the pandemic in many countries, as well as the slow process which is demanded to obtain the final approval or delays in the processes, as seen in 2016 [12], for example. In the same way, this year, a few drugs could have had an expected approval deadline in 2022, but, due to delays, perhaps they are going to be approved in 2023. This decrease in authorizations should not be interpreted as a greater failure on the part of the pharmaceutical industry, as authorization has been granted to key drugs and there has been a trend to devote resources to an increasing number of rare diseases and conditions.

It should be noted that the decrease in the approvals in 2022 only applied to the small molecules category, not to Biologics. In this regard, 15 Biologics have been approved in 2022. In terms of the period 2015–2022, this year is among those with the highest number of biopharmaceuticals to receive the green light [1,30].

Special mention is given to four Biologics: the first bispecific fusion protein approved to date, namely Kimmtrak^TM^ (tebentafusp), and the antibodies Vabysmo^TM^ (faricimab), Tecvayli^TM^ (teclistamab), and Lunsumio^TM^ (mosunetuzumab), two of which are Orphan Drugs. This harvest makes 2022 the year with the highest number of bispecific biologic products to receive authorization. Of note, Vabysmo^TM^ is produced by the giant Genentech, which also manufactures two of the Biologics approved in 2022. This company has received authorization for a Biologic almost every year between 2015 and 2022. Indeed, in 2017, three of its Biologics received the green light.

Although cancer continues to be the most targeted disease for Biologics, only six out of the fifteen drugs authorized in 2022 are indicated for cancer. This may indicate a trend towards targeting other diseases. The harvest of 2022 included an ADC, Elahere^TM^, which, like all the other ADCs approved to date, is indicated for cancer. However, it carries a distinct payload from previous ADCs. As in previous years, in 2022, autoimmune conditions continued to be the second in the ranking of targets after cancer, with four out of the fifteen Biologics authorized for this indication.

For aesthetic purposes, 2022 saw the approval of Daxxify^TM^ (daxibotulinumtoxin A), the effects of which proved to have a longer duration than those of the widely used onabotulinumtoxin A. Of note, the last botulinum toxin to be approved, prabotulinumtoxin A, was in 2019. It could be speculated that there is an emerging trend to provide alternatives to Botox^TM^. Daaxify^TM^ falls into the natural product section, along with NexoBrid^TM^ (anacaulase), a mixture of enzymes from pineapple [30,79,80,94]. Regarding the efficacy of the other Biologics in 2022, so far, data show that they were all either superior to previous Biologics or non-inferior.

Each year brings approvals for new targets and 2022 was no different. In this regard, important advancements were made, such as Kimmtrak^TM^ (tebentafusp). In addition to being the first bispecific fusion protein, it is the first Biologic indicated for the treatment of unresectable or metastatic uveal melanoma. Furthermore, Spevigo^TM^ (spesolimab) is the first mAb to specifically treat generalized pustular psoriasis flares [19,20]. Both drugs have been granted Orphan Drug Status by the FDA. 

In the context of the increasing concern regarding antibiotic resistance worldwide, the pharmaceutical industry appears to be struggling to develop antibacterial Biologics. This is reflected by the fact that only two such products have been authorized since 2015 [1]. The complexity of bacteria and the presence of polymicrobial infections may hinder the development of such Biologics. In this regard, for example, while mAbs are highly selective drugs that are directed at one specific target, they may not be effective against the extremely high number of possible targets on bacterial surfaces that appear to be involved in infection. Such features make the development of new drugs even harder and greater research efforts will be needed [15]. The combination of mAbs or bispecific mAbs emerges as a potentially relevant approach when addressing multiple targets in bacteria.

## 6. Conclusions

Notable advancements in the Biologics market were witnessed between 2015 and 2021, and 2022 was no different. Despite the smaller number of approvals than in previous years, the total number for all Biologics did not vary from previous years. In this regard, the decrease applied only to NCE.

Some of the drugs authorized in 2022 aimed at diseases and conditions without a specific standard treatment, and have novel mechanisms of action. This finding reflects the continued efforts to tackle challenges and provide patients with diseases other than cancer with more treatment options. Given all the ongoing trials found for the Biologics presented herein, it is observed that a great effort to repurpose these Biologics is in order to find other therapeutic indications for them, but it is still too early to find results posted on clinicaltrials.gov.

The FDA granted Orphan Drug Status to seven out of the fifteen Biologics approved in 2022. This figure emphasizes the tendency of the pharmaceutical industry to embrace the important fight against rare diseases and conditions.

Given all the Orphan Drug Statuses granted, plus the fact that cancer remains the main targeted disease, followed by autoimmune conditions, we wonder whether or not there is a tendency for the Biologics market to pay more attention to those and set aside all the other diseases and conditions that exist which also need support; for example, the antimicrobial resistance mentioned.

## Figures and Tables

**Table 1:** Correlation of Total Drug Approvals vs. Biologics Approvals by the FDA.

Year	Total Drugs Approved (Biologics and NCEs)	Biologics Approved	References
2022	37	15 (40.5%)	[3]
2021	50	14 (28%)	[4]
2020	53	15 (28.3%)	[5]
2019	48	12 (25%)	[6]
2018	59	17 (28.8%)	[7]
2017	46	13 (28.2%)	[8]
2016	22	7 (31.8%)	[9]
2015	45	13 (28.8%)	[10]

**Table 2:** Orphan Drug Status granted by the FDA from 2015 to 2022.

Year	Biologics Approved	Orphan Drug Status Granted for New Biologics	References
2015	13	7 (53%)	[10]
2016	7	2 (28%)	[9]
2017	13	5 (38%)	[8]
2018	17	13 (76%)	[7]
2019	12	9 (75%)	[6]
2020	15	10 (66%)	[5]
2021	14	7 (50%)	[4]
2022	15	7 (46%)	[3]

**Table 3:** Biologics for cancer approved by the Food and Drug Administration in 2022.

Trade Name and Active Ingredient	Class	Target/Mechanism of Action	Original Approval Date	Manufacturer	Therapeutic Indication
Kimmtrak^TM^ (tebentafusp) ^1^ [3,19,20]	Bispecific Fusion Protein	TCR arm binds to gp100 on Uveal Melanoma cells and the anti-CD3 effector binds to T Lymphocytes	25 January 2022	Immunocore Limited	Unresectable or Metastatic Uveal Melanoma
Opdualag^TM^ (nivolumab and relatlimab) ^1^ [3,21]	Combination of Human mAbs	Blocks LAG-3 and PD-1 receptors from binding to their ligands	18 March 2022	Bristol-Myers Squibb Company	Unresectable or Metastatic Melanoma
Imjudo^TM^ (tremelimumab) [3,22,23]	Human mAb	Binds to CTLA-4	21 October 2022	AstraZeneca AB	uHCC and metastatic NSCLC
Tecvayli^TM^ (teclistamab) ^1^ [3,24]	Bispecific mAb	CD-3 receptor and BCMA	25 October 2022	Janssen Biotech, Inc.	Multiple Myeloma
Elahere^TM^ (mirvetuximab soravtansine) [3,25]	ADC (chimeric mAb)	IgG1 directed against FRα, releasing DM4	14 November 2022	ImmunoGen, Inc.	PROC, PPC, or Fallopian Tube Cancer
Lunsumio^TM^ (mosunetuzumab) [26,27]	Bispecific mAb	Binds to CD20 and CD3 receptors	22 December 2022	Genentech, Inc.	Relapsed or Refractory Follicular Lymphoma

^1^—Orphan Drug; TCR—T-Cell Receptor; gp100—Glycoprotein 100; CD—Cluster of Differentiation; LAG-3—Lymphocyte Activation Gene-3; PD-1—Programmed Death Receptor-1; CTLA-4—Cytotoxic T-Lymphocyte-Associated Antigen 4; uHCC—Unresectable Hepatocellular Carcinoma; NSCLC—Non-Small Cell Lung Cancer; BCMA—B-Cell Maturation Antigen; ADC—Antibody-Drug Conjugate; IgG1—Immunoglobulin G 1; DM4—Antitubulin Agent; FRα—Folate Receptor α; PROC—Platinum-Resistant Epithelial Ovarian Cancer; and PPC—Primary Peritoneal Cancer.

**Table 4:** Biologics approved for autoimmune conditions by the Food and Drug Administration in 2022.

Trade Name and Active Ingredient	Class	Target/Mechanism of Action	Original Approval Date	Manufacturer	Therapeutic Indication
Enjaymo^TM^ (sutimlimab) ^1^ [3,56]	Humanized mAb	Inhibits the complement pathway and binds to complement protein component 1	4 February 2022	Bioverativ Therapeutics, Inc.	Decrease the need of RBC transfusion due to hemolysis in CAD
Spevigo^TM^ (spesolimab) ^1^ [3,19,20]	Humanized mAb	IL36R Antagonist	1 September 2022	Boehringer Ingelheim Pharmaceuticals, Inc.	Generalized Pustular Psoriasis Flares
Tzield^TM^ (teplizumab) [3,57,58]	Humanized mAb	Binds to CD3	18 November 2022	Provention Bio, Inc	Delay the onset of Stage 2 or 3 Type 1 Diabetes
Briumvi^TM^ (ublituximab) [59]	Chimeric mAb	Binds to CD20 on B-Cells	28 December 2022	TG Therapeutics, Inc.	Multiple Sclerosis

^1^—Orphan Drug; RBC—Red Blood Cell; CAD—Cold Agglutinin Disease; IL36R—Interleukin-36 Receptor; CD—Cluster of Differentiation.

**Table 5:** Botulinum Toxin A approved by the Food and Drug Administration in 2022.

Trade Name and Active Ingredient	Class	Target/Mechanism of Action	Original Approval Date	Manufacturer	Therapeutic Indication
Daxxify^TM^ (daxibotulinum- toxin A) [3,81]	Botulinum toxin A (protein-based therapy)	Inhibits the release of Acetylcholine to the Neuromuscular Junction	7 September 2022	Revance Therapeutics, Inc.	Improve glabellar lines associated with corrugator and/or procerus muscle activity

**Table 6:** Biologic for eye disorders approved by the Food and Drug Administration in 2022.

Trade Name and Active Ingredient	Class	Target/Mechanism of Action	Original Approval Date	Manufacturer	Therapeutic Indication
Vabysmo^TM^ (faricimab) [3,35]	Bispecific mAb	VEGF-A and Ang-2	28 January 2022	Genentech, Inc.	nAMD and DME

nAMD—Neovascular (Wet) Age-Related Macular Degeneration; DME—Diabetic Macular Edema; VEGF—Vascular Endothelial Growth Factor; Ang-2—Angiopoietin-2.

**Table 7:** Proteins and enzymes approved by the Food and Drug Administration in 2022.

Trade Name and Active Ingredient	Class	Target/Mechanism of Action	Original Approval Date	Manufacturer	Therapeutic Indication
Xenpozyme^TM^ (olipudase alfa) ^1^ [3,91,92]	Enzyme	ASM replacement therapy, reducing SM accumulation	31 August 2022	Genzyme Corporation	ASMD
Rolvedon^TM^ (eflapegrastim) [3,93]	rhG-CSF combined with an FC Fragment of human IgG4	Binds to G-CSF receptors	9 September 2022	Spectrum Pharmaceuticals, Inc.	Decrease the incidence of infection, as manifested by chemotherapy-induced Neutropenia
NexoBrid^TM^ (anacaulase) ^1^ [94]	Compound of Enzymes	Dissolves burn wound eschars	28 December 2022	MediWound, Ltd.	Schar removal with partial- or full-thickness thermal burns

^1^—Orphan Drug; ASM—Acid Sphingomyelinase; ASMD—Acid Sphingomyelinase Deficiency; SM—Sphingomyelin; rhG-CSF—Recombinant Human Granulocyte Colony-Stimulating Factor; G-CSF—Granulocyte Colony-Stimulating Factor.

## Data Availability

Not applicable.

## References

[1] Martins A.C., Oshiro M.Y., Albericio F., de la Torre B.G., Pereira G.J.V., Gonzaga R.V. (2022). Trends and Perspectives of Biological Drug Approvals by the FDA: A Review from 2015 to 2021. Biomedicines.

[2] Torre B., Albericio F. (2017). The Pharmaceutical Industry in 2016. An Analysis of FDA Drug Approvals from a Perspective of the Molecule Type. Molecules.

[3] U.S. Food & Drug Administration Novel Drug Approvals for 2022. https://www.fda.gov/drugs/new-drugs-fda-cders-new-molecular-entities-and-new-therapeutic-biological-products/novel-drug-approvals-2022.

[4] U.S. Food & Drug Administration Novel Drug Approvals for 2021. https://www.fda.gov/drugs/new-drugs-fda-cders-new-molecular-entities-and-new-therapeutic-biological-products/novel-drug-approvals-2021.

[5] U.S. Food & Drug Administration Novel Drug Approvals for 2020. https://www.fda.gov/drugs/new-drugs-fda-cders-new-molecular-entities-and-new-therapeutic-biological-products/novel-drug-approvals-2020.

[6] U.S. Food & Drug Administration Novel Drug Approvals for 2019. https://www.fda.gov/drugs/new-drugs-fda-cders-new-molecular-entities-and-new-therapeutic-biological-products/novel-drug-approvals-2019.

[7] U.S. Food & Drug Administration Novel Drug Approvals for 2018. https://www.fda.gov/drugs/new-drugs-fda-cders-new-molecular-entities-and-new-therapeutic-biological-products/novel-drug-approvals-2018.

[8] U.S. Food & Drug Administration Novel Drug Approvals for 2017. https://www.fda.gov/drugs/new-drugs-fda-cders-new-molecular-entities-and-new-therapeutic-biological-products/novel-drug-approvals-2017.

[9] U.S. Food & Drug Administration Novel Drug Approvals for 2016. https://www.fda.gov/drugs/new-drugs-fda-cders-new-molecular-entities-and-new-therapeutic-biological-products/novel-drug-approvals-2016.

[10] U.S. Food and Drug Administration Novel Drug Approvals for 2015. https://www.fda.gov/drugs/new-drugs-fda-cders-new-molecular-entities-and-new-therapeutic-biological-products/novel-drug-approvals-2015.

[11] Ledford H. US Drug Approvals Plummet in 2016. https://www.nature.com/articles/nature.2016.21192.

[12] Mullard A. (2017). 2016 FDA Drug Approvals. Nat. Rev. Drug Discov..

[13] U.S. Food & Drug Administration FDA’s Work to Combat the COVID-19 Pandemic. https://www.fda.gov/media/160998/download.

[14] World Health Organization Global Action Plan on Antimicrobial Resistance. https://apps.who.int/iris/bitstream/handle/10665/193736/9789241509763_eng.pdf?sequence=1&isAllowed=y.

[15] Zurawski D.V., McLendon M.K. (2020). Monoclonal Antibodies as an Antibacterial Approach Against Bacterial Pathogens. Antibiotics.

[16] U.S. Food & Drug Administration Designating an Orphan Product: Drugs and Biological Products. https://www.fda.gov/industry/medical-products-rare-diseases-and-conditions/designating-orphan-product-drugs-and-biological-products#:~:text=The%20FDA%20has%20authority%20to,Exemption%20from%20user%20fees.

[17] Benedetto Tiz D., Bagnoli L., Rosati O., Marini F., Sancineto L., Santi C. (2023). Top Selling (2026) Small Molecule Orphan Drugs: A Journey into Their Chemistry. Int. J. Mol. Sci..

[18] Fonseca D.A., Amaral I., Pinto A.C., Cotrim M.D. (2019). Orphan Drugs: Major Development Challenges at the Clinical Stage. Drug Discov. Today.

[19] U.S. Food & Drug Administration FDA Approves Tebentafusp-Tebn for Unresectable or Metastatic Uveal Melanoma. https://www.fda.gov/drugs/resources-information-approved-drugs/fda-approves-tebentafusp-tebn-unresectable-or-metastatic-uveal-melanoma.

[20] Dhillon S. (2022). Tebentafusp: First Approval. Drugs.

[21] Tawbi H.A., Schadendorf D., Lipson E.J., Ascierto P.A., Matamala L., Castillo Gutiérrez E., Rutkowski P., Gogas H.J., Lao C.D., de Menezes J.J. (2022). Relatlimab and Nivolumab versus Nivolumab in Untreated Advanced Melanoma. N. Engl. J. Med..

[22] U.S. Food & Drug Administration FDA Approves Tremelimumab in Combination with Durvalumab and Platinum-Based Chemotherapy for Metastatic Non-Small Cell Lung Cancer. https://www.fda.gov/drugs/resources-information-approved-drugs/fda-approves-tremelimumab-combination-durvalumab-and-platinum-based-chemotherapy-metastatic-non.

[23] U.S. Food and Drug Administration IMJUDO® (Tremelimumab-Actl) Injection, for Intravenous Use Initial U.S. Approval: 2022. https://www.accessdata.fda.gov/drugsatfda_docs/label/2022/761289lbl.pdf.

[24] Kang C. (2022). Teclistamab: First Approval. Drugs.

[25] U.S. Food and Drug Administration ELAHERE^TM^ (Mirvetuximab Soravtansine-Gynx) Injection, for Intravenous Use Initial U.S. Approval: 2022. https://www.accessdata.fda.gov/drugsatfda_docs/label/2022/761310s000lbl.pdf.

[26] U.S. Food and Drug Administration LUNSUMIO^TM^ (Mosunetuzumab-Axgb) Injection, for Intravenous Use Initial U.S. Approval: 2022. https://www.accessdata.fda.gov/drugsatfda_docs/label/2022/761263s000lbl.pdf.

[27] U.S. Food and Drug Administration FDA Grants Accelerated Approval to Mosunetuzumab-Axgb for Relapsed or Refractory Follicular Lymphoma. https://www.fda.gov/drugs/resources-information-approved-drugs/fda-grants-accelerated-approval-mosunetuzumab-axgb-relapsed-or-refractory-follicular-lymphoma.

[28] Twomey J.D., Zhang B. (2021). Cancer Immunotherapy Update: FDA-Approved Checkpoint Inhibitors and Companion Diagnostics. AAPS J..

[29] Nathan P., Hassel J.C., Rutkowski P., Baurain J.-F., Butler M.O., Schlaak M., Sullivan R.J., Ochsenreither S., Dummer R., Kirkwood J.M. (2021). Overall Survival Benefit with Tebentafusp in Metastatic Uveal Melanoma. N. Engl. J. Med..

[30] de la Torre B.G., Albericio F. (2023). The Pharmaceutical Industry in 2022: An Analysis of FDA Drug Approvals from the Perspective of Molecules. Molecules.

[31] Fanale M.A., Younes A. (2007). Monoclonal Antibodies in the Treatment of Non-Hodgkin’s Lymphoma. Drugs.

[32] Melaragno A. (2017). Rituximab/Hyaluronidase (Rituxan Hycela^TM^). Oncol. Times.

[33] Scott L.J., Kim E.S. (2018). Emicizumab-Kxwh: First Global Approval. Drugs.

[34] Syed Y.Y. (2021). Amivantamab: First Approval. Drugs.

[35] Chaplin S. (2022). Faricimab for Treating Wet AMD and Diabetic Macular Oedema. Prescriber.

[36] Kudo M. (2022). Durvalumab plus Tremelimumab in Unresectable Hepatocellular Carcinoma. Hepatobiliary Surg. Nutr..

[37] Moreau P., Garfall A.L., van de Donk N.W.C.J., Nahi H., San-Miguel J.F., Oriol A., Nooka A.K., Martin T., Rosinol L., Chari A. (2022). Teclistamab in Relapsed or Refractory Multiple Myeloma. N. Engl. J. Med..

[38] Janssen Research & Development, LLC A Study of Teclistamab in Participants with Relapsed or Refractory Multiple Myeloma (MajesTEC-1). https://clinicaltrials.gov/ct2/show/NCT04557098.

[39] Janssen Research & Development, LLC Dose Escalation Study of Teclistamab, a Humanized BCMA*CD3 Bispecific Antibody, in Participants with Relapsed or Refractory Multiple Myeloma (MajesTEC-1). https://clinicaltrials.gov/ct2/show/NCT03145181.

[40] U.S. Food and Drug Administration FDA Grants Accelerated Approval to Mirvetuximab Soravtansine-Gynx for FRα Positive, Platinum-Resistant Epithelial Ovarian, Fallopian Tube, or Peritoneal Cancer. https://www.fda.gov/drugs/resources-information-approved-drugs/fda-grants-accelerated-approval-mirvetuximab-soravtansine-gynx-fra-positive-platinum-resistant.

[41] ImmunoGen, Inc A Study of Mirvetuximab Soravtansine in Platinum-Resistant, Advanced High-Grade Epithelial Ovarian, Primary Peritoneal, or Fallopian Tube Cancers with High Folate Receptor-Alpha Expression (SORAYA). https://clinicaltrials.gov/ct2/show/NCT04296890?term=NCT04296890&draw=2&rank=1.

[42] Immunocore Ltd Phase 1b/2 Study of the Combination of IMCgp100 with Durvalumab and/or Tremelimumab in Advanced Cutaneous Melanoma. https://clinicaltrials.gov/ct2/show/NCT02535078?term=tebentafusp&draw=2&rank=4.

[43] Jonsson Comprehensive Cancer Center Nivolumab and Relatlimab in Treating Participants with Advanced Chordoma. https://clinicaltrials.gov/ct2/show/NCT03623854?term=nivolumab+and+relatlimab&draw=2&rank=4.

[44] Sidney Kimmel Comprehensive Cancer Center at Johns Hopkins Study of Nivolumab and Relatlimab in Patients with Microsatellite Stable (MSS) Advanced Colorectal Cancer. https://clinicaltrials.gov/ct2/show/NCT03642067?term=nivolumab+and+relatlimab&draw=2&rank=5.

[45] Bristol-Myers Squibb A Study of Nivolumab and Relatlimab in Combination with Bevacizumab in Advanced Liver Cancer (RELATIVITY-106). https://clinicaltrials.gov/ct2/show/NCT05337137?term=nivolumab+and+relatlimab&draw=2&rank=7.

[46] Jose Lutzky M.D. Nivolumab Plus Relatlimab in Patients with Metastatic Uveal Melanoma. https://clinicaltrials.gov/ct2/show/NCT04552223?term=nivolumab+and+relatlimab&draw=2&rank=8.

[47] Peter Black P.U. Trial of Local Cystoscopic Injection of Tremelimumab Plus Systemic Durvalumab for High Risk Non-Muscle Invasive Bladder Cancer (Rideau). https://clinicaltrials.gov/ct2/show/NCT05120622?term=tremelimumab&draw=2&rank=1.

[48] AstraZeneca Durvalumab and Tremelimumab for Pediatric Malignancies. https://clinicaltrials.gov/ct2/show/NCT03837899?term=tremelimumab&draw=2&rank=2.

[49] Stephen Hodi F. Phase I Clinical Trial of Tremelimumab Plus MEDI3617 in Patients with Unresectable Stage III or Stage IV Melanoma. https://clinicaltrials.gov/ct2/show/NCT02141542?term=tremelimumab&draw=2&rank=7.

[50] M.D. Anderson Cancer Center Mirvetuximab Soravtansine as First Line in Treating Patients with Triple Negative Breast Cancer. https://clinicaltrials.gov/ct2/show/NCT03106077?term=mirvetuximab+soravtansine&draw=2&rank=4.

[51] Konstantinopoulos P., Dana-Farber Cancer Institute A Phase 2 Study of Mirvetuximab Soravtansine (IMGN853) and Pembrolizumab in Endometrial Cancer (EC). https://clinicaltrials.gov/ct2/show/NCT03835819?term=Mirvetuximab+Soravtansine&draw=2&rank=9.

[52] Lazaros Lekakis A.P. CAR-T Cell Therapy, Mosunetuzumab and Polatuzumab for Treatment of Refractory/Relapsed Aggressive Non-Hodgkin’s Lymphoma (NHL). https://clinicaltrials.gov/ct2/show/NCT05260957.

[53] Washington University School of Medicine Mosunetuzumab Consolidation Therapy after AutoSCT in r/r Aggressive B Cell Lymphoma. https://clinicaltrials.gov/ct2/show/NCT05412290?term=mosunetuzumab&draw=2&rank=1.

[54] Roche H.-L. A Study Evaluating the Safety, Efficacy, and Pharmacokinetics of Mosunetuzumab in Patients with Relapsed or Refractory Chronic Lymphocytic Leukemia. https://clinicaltrials.gov/ct2/show/NCT05091424.

[55] Roche H.-L. A Study to Evaluate the Safety, Tolerability, Pharmacokinetics, and Pharmacodynamics of Subcutaneously Administered Mosunetuzumab to Participants with Systemic Lupus Erythematosus. https://clinicaltrials.gov/ct2/show/NCT05155345?term=mosunetuzumab&draw=2&rank=5.

[56] Dhillon S. (2022). Sutimlimab: First Approval. Drugs.

[57] Hirsch J.S. (2022). FDA Approves Teplizumab: A Milestone in Type 1 Diabetes. Lancet Diabetes Endocrinol..

[58] U.S. Food and Drug Administration TZIELD^TM^ (Teplizumab-Mzwv) Injection, for Intravenous Use Initial U.S. Approval: 2022. https://www.accessdata.fda.gov/drugsatfda_docs/label/2022/761183s000lbl.pdf.

[59] U.S. Food and Drug Administration BRIUMVI^TM^ (Ublituximab-Xiiy) Injection, for Intravenous Use Initial U.S. Approval: 2022. https://www.accessdata.fda.gov/drugsatfda_docs/label/2022/761238s000lbl.pdf.

[60] Gooderham M.J., van Voorhees A.S., Lebwohl M.G. (2019). An Update on Generalized Pustular Psoriasis. Expert Rev. Clin. Immunol..

[61] Bachelez H., Choon S.-E., Marrakchi S., Burden A.D., Tsai T.-F., Morita A., Turki H., Hall D.B., Shear M., Baum P. (2019). Inhibition of the Interleukin-36 Pathway for the Treatment of Generalized Pustular Psoriasis. N. Engl. J. Med..

[62] Ingelheim B. BI655130 Single Dose in Generalized Pustular Psoriasis. https://clinicaltrials.gov/ct2/show/NCT02978690.

[63] U.S. Food and Drug Administration OCREVUSTM (Ocrelizumab) Injection, for Intravenous Use Initial U.S. Approval: 2017. https://www.accessdata.fda.gov/drugsatfda_docs/label/2017/761053lbl.pdf.

[64] Steinman L., Fox E., Hartung H.-P., Alvarez E., Qian P., Wray S., Robertson D., Huang D., Selmaj K., Wynn D. (2022). Ublituximab versus Teriflunomide in Relapsing Multiple Sclerosis. N. Engl. J. Med..

[65] U.S. Food and Drug Administration FDA Approves First Drug That Can Delay Onset of Type 1 Diabetes. https://www.fda.gov/news-events/press-announcements/fda-approves-first-drug-can-delay-onset-type-1-diabetes.

[66] U.S. Food and Drug Administration ENJAYMO^TM^ (Sutimlimab-Jome) Injection, for Intravenous Use Initial U.S. Approval: 2022. https://www.accessdata.fda.gov/drugsatfda_docs/label/2022/761164s000lbl.pdf.

[67] U.S. Food and Drug Administration FDA Approves Treatment for Adults with Rare Type of Anemia. https://www.fda.gov/drugs/news-events-human-drugs/fda-approves-treatment-adults-rare-type-anemia.

[68] Röth A., Barcellini W., D’Sa S., Miyakawa Y., Broome C.M., Michel M., Kuter D.J., Jilma B., Tvedt T.H.A., Fruebis J. (2021). Sutimlimab in Cold Agglutinin Disease. N. Engl. J. Med..

[69] Mrowietz U., Burden A.D., Pinter A., Reich K., Schäkel K., Baum P., Datsenko Y., Deng H., Padula S.J., Thoma C. (2021). Spesolimab, an Anti-Interleukin-36 Receptor Antibody, in Patients with Palmoplantar Pustulosis: Results of a Phase IIa, Multicenter, Double-Blind, Randomized, Placebo-Controlled Pilot Study. Dermatol. Ther..

[70] Ingelheim B. A Study to Test How Effective and Safe Different Doses of BI 655130 Are in Patients with a Moderate to Severe Form of the Skin Disease Palmoplantar Pustulosis. https://beta.clinicaltrials.gov/study/NCT04015518?distance=50&term=spesolimab&rank=1.

[71] Ingelheim B. A Study Investigating Long-Term Treatment with Spesolimab in People with a Skin Disease Called Hidradenitis Suppurativa Who Completed a Previous Clinical Trial. https://beta.clinicaltrials.gov/study/NCT04876391?distance=50&term=spesolimab&rank=6.

[72] Ingelheim B. BI655130 (SPESOLIMAB) Induction Treatment in Patients with Moderate-to-Severe Ulcerative Colitis. https://clinicaltrials.gov/ct2/show/study/NCT03482635?term=spesolimab&draw=3&rank=13.

[73] Ingelheim B. A Study to Test Whether Spesolimab Helps People with Crohn’s Disease Who Have Symptoms of Bowel Obstruction. https://clinicaltrials.gov/ct2/show/NCT05013385?term=spesolimab&draw=2&rank=5.

[74] Ingelheim B. A Study in Patients with Atopic Eczema to Test How Effective BI 655130 Is and How Well It Is Tolerated. https://clinicaltrials.gov/ct2/show/NCT03822832?term=spesolimab&draw=4&rank=21.

[75] Iznardo H., Puig L. (2022). IL-1 Family Cytokines in Inflammatory Dermatoses: Pathogenetic Role and Potential Therapeutic Implications. Int. J. Mol. Sci..

[76] Weill Medical College of Cornell University Umbralisib Plus Ublituximab (U2) in Progressive CLL After Novel Therapy. https://clinicaltrials.gov/ct2/show/NCT04149821?term=ublituximab&recrs=ab&draw=2&rank=2.

[77] City of Hope Medical Center Tazemetostat in Combination with Umbralisib and Ublituximab for the Treatment Relapsed or Refractory Follicular Lymphoma. https://clinicaltrials.gov/ct2/show/NCT05152459?term=ublituximab&recrs=ab&draw=2&rank=3.

[78] Bertucci V., Solish N., Kaufman-Janette J., Yoelin S., Shamban A., Schlessinger J., Snyder D., Gallagher C., Liu Y., Shears G. (2020). DaxibotulinumtoxinA for Injection Has a Prolonged Duration of Response in the Treatment of Glabellar Lines: Pooled Data from Two Multicenter, Randomized, Double-Blind, Placebo-Controlled, Phase 3 Studies (SAKURA 1 and SAKURA 2). J. Am. Acad. Dermatol..

[79] Bertucci V., Humphrey S., Carruthers J., Solish N., Muhn C., Swift A., Rubio R.G., Shears G., Rosen N. (2017). Comparing Injectable DaxibotulinumtoxinA and OnabotulinumtoxinA in Moderate and Severe Glabellar Lines: Additional Analyses From a Phase 2, Randomized, Dose-Ranging, Double-Blind, Multicenter Study. Dermatol. Surg..

[80] Solish N., Carruthers J., Kaufman J., Rubio R.G., Gross T.M., Gallagher C.J. (2021). Overview of DaxibotulinumtoxinA for Injection: A Novel Formulation of Botulinum Toxin Type A. Drugs.

[81] U.S. Food and Drug Administration DAXXIFY^TM^ (DaxibotulinumtoxinA-Lanm) for Injection, for Intramuscular Use Initial U.S. Approval: 2022. https://www.accessdata.fda.gov/drugsatfda_docs/label/2022/761127s000lbl.pdf.

[82] Revance Therapeutics, Inc Efficacy and Safety of Botulinum Toxin Type A for Injection to Treat Glabellar Lines. https://www.clinicaltrials.gov/ct2/show/NCT02303002?term=NCT02303002&draw=2&rank=1.

[83] Brundridge W.L., Czyz C.N., Foster J.A., DeBacker C.M., Holck D.E.E. (2021). Comparison of Prabotulinumtoxin A to Onabotulinumtoxin A in the Treatment of Lateral Canthal Rhytids: A Side-by-Side, Randomized, Double-Blind Comparison. Am. J. Cosmet. Surg..

[84] Markham A. (2019). Brolucizumab: First Approval. Drugs.

[85] Finger R.P., Dennis N., Freitas R., Quenéchdu A., Clemens A., Karcher H., Souied E.H. (2022). Comparative Efficacy of Brolucizumab in the Treatment of Neovascular Age-Related Macular Degeneration: A Systematic Literature Review and Network Meta-Analysis. Adv. Ther..

[86] Shirley M. (2022). Faricimab: First Approval. Drugs.

[87] Khanani A.M., Patel S.S., Ferrone P.J., Osborne A., Sahni J., Grzeschik S., Basu K., Ehrlich J.S., Haskova Z., Dugel P.U. (2020). Efficacy of Every Four Monthly and Quarterly Dosing of Faricimab vs Ranibizumab in Neovascular Age-Related Macular Degeneration. JAMA Ophthalmol..

[88] Greater Houston Retina Research Safety and Efficacy of Faricimab in Patients with NPDR (MAGIC). https://clinicaltrials.gov/ct2/show/NCT05681884?term=faricimab&draw=2&rank=4.

[89] Roche H.-L. A Study to Evaluate the Efficacy and Safety of Faricimab in Participants with Macular Edema Secondary to Branch Retinal Vein Occlusion (BALATON). https://clinicaltrials.gov/ct2/show/NCT04740905?term=faricimab&draw=4&rank=7.

[90] Roche H.-L. A Study to Evaluate the Efficacy and Safety of Faricimab in Participants with Macular Edema Secondary to Central Retinal or Hemiretinal Vein Occlusion (COMINO). https://clinicaltrials.gov/ct2/show/NCT04740931?term=faricimab&draw=2&rank=8.

[91] Keam S.J. (2022). Olipudase Alfa: First Approval. Drugs.

[92] U.S. Food & Drug Administration FDA Approves First Treatment for Acid Sphingomyelinase Deficiency, a Rare Genetic Disease. https://www.fda.gov/news-events/press-announcements/fda-approves-first-treatment-acid-sphingomyelinase-deficiency-rare-genetic-disease.

[93] U.S. Food and Drug Administration ROLVEDONTM (Eflapegrastim-Xnst) Injection, for Subcutaneous Use Initial U.S. Approval: 2022. https://www.accessdata.fda.gov/drugsatfda_docs/label/2022/761148Orig1s000Corrected_lbl.pdf.

[94] U.S. Food and Drug Administration NEXOBRID^®^ (Anacaulase-Bcdb) for Topical Gel Initial U.S. Approval: 2022. https://www.accessdata.fda.gov/drugsatfda_docs/label/2023/761192s000lbl.pdf.

[95] Pinto C., Sousa D., Ghilas V., Dardis A., Scarpa M., Macedo M. (2021). Acid Sphingomyelinase Deficiency: A Clinical and Immunological Perspective. Int. J. Mol. Sci..

[96] Wasserstein M.P., Desnick R.J., Schuchman E.H., Hossain S., Wallenstein S., Lamm C., McGovern M.M. (2004). The Natural History of Type B Niemann-Pick Disease: Results From a 10-Year Longitudinal Study. Pediatrics.

[97] Genzyme, a Sanofi Company Efficacy, Safety, Pharmacodynamic, and Pharmacokinetics Study of Olipudase Alfa in Patients with Acid Sphingomyelinase Deficiency (ASCEND). https://clinicaltrials.gov/ct2/show/results/NCT02004691?term=olipudase+alfa&draw=2&rank=4.

[98] Wasserstein M., Lachmann R., Hollak C., Arash-Kaps L., Barbato A., Gallagher R.C., Giugliani R., Guelbert N.B., Ikezoe T., Lidove O. (2022). A Randomized, Placebo-Controlled Clinical Trial Evaluating Olipudase Alfa Enzyme Replacement Therapy for Chronic Acid Sphingomyelinase Deficiency (ASMD) in Adults: One-Year Results. Genet. Med..

[99] Spectrum Pharmaceuticals Inc. SPI-2012 vs. Pegfilgrastim in Management of Neutropenia in Breast Cancer Participants with Docetaxel and Cyclophosphamide. https://clinicaltrials.gov/ct2/show/NCT02953340?term=NCT02953340&draw=2&rank=1.

[100] Spectrum Pharmaceuticals Inc. SPI-2012 vs. Pegfilgrastim in the Management of Neutropenia in Participants with Breast Cancer with Docetaxel and Cyclophosphamide (ADVANCE) (ADVANCE). https://clinicaltrials.gov/ct2/show/NCT02643420?term=NCT02643420&draw=2&rank=1.

[101] Cobb P.W., Moon Y.W., Mezei K., Láng I., Bhat G., Chawla S., Hasal S.J., Schwartzberg L.S. (2020). A Comparison of Eflapegrastim to Pegfilgrastim in the Management of Chemotherapy-induced Neutropenia in Patients with Early-stage Breast Cancer Undergoing Cytotoxic Chemotherapy (RECOVER): A Phase 3 Study. Cancer Med..

[102] Hirche C., Citterio A., Hoeksema H., Koller J., Lehner M., Martinez J.R., Monstrey S., Murray A., Plock J.A., Sander F. (2017). Eschar Removal by Bromelain Based Enzymatic Debridement (Nexobrid^®^) in Burns: An European Consensus. Burns.

[103] Spectrum Pharmaceuticals Inc. A Study to Evaluate the Safety and Pharmacokinetics of Eflapegrastim in Pediatric Participants with Solid Tumors or Lymphomas and Treated with Myelosuppressive Chemotherapy. https://clinicaltrials.gov/ct2/show/NCT04570423?term=eflapegrastim&draw=2&rank=1.

[104] Spectrum Pharmaceuticals Inc. Open-Label, Phase 1 Study to Evaluate Duration of Severe Neutropenia after Same-Day Dosing of Eflapegrastim in Patients with Breast-Cancer. https://clinicaltrials.gov/ct2/show/NCT04187898?term=eflapegrastim&draw=2&rank=2.

